# Preparation of porous biochar from fusarium wilt-infected banana straw for remediation of cadmium pollution in water bodies

**DOI:** 10.1038/s41598-024-63954-4

**Published:** 2024-06-15

**Authors:** Chengxiang Gao, Yi Lan, Yaowei Zhan, Yuechen Li, Jiaquan Jiang, Yuanqiong Li, Lidan Zhang, Xiaolin Fan

**Affiliations:** 1 Guangdong Engineering Technology Research Center of Low Carbon Agricultural Green Inputs, South China Agricultural University, Guangzhou City, 510642 China; 2 R&D Center of Environmental Friendly Fertilizer Science and Technology of Education Department of Guangdong Province, College of Natural Resources and Environment, South China Agricultural University, Guangzhou City, 510642 China

**Keywords:** Banana straw, Pathogen viability, KOH modified biohchar, Cadmium adsorption, Precipitation and complexation, Pollution remediation, Agroecology

## Abstract

The problem of cadmium pollution and its control is becoming increasingly severe issue in the world. Banana straw is an abundant bio raw material, but its burning or discarding in field not only causes pollution but also spreads fusarium wilt. The objective of this paper is to utilize biochar derived from the wilt-infected banana straw for remediation of Cd(II) pollution while to eliminate the pathogen. The activity of wilt pathogen in biochar was determined by PDA petri dish test. The Cd(II) adsorption of the biochar was determined by batch adsorption experiments. The effects of KOH concentration (0.25, 0.5 and 0.75 M) on the physicochemical characteristics of the biochar were also observed by BET, SEM, FTIR, XRD and XPS. Results showed that pristine banana straw biochar (PBBC) did not harbor any pathogen. The specific surface area (SSA) and Cd(II) adsorption capacity of 0.75 M KOH modified banana straw biochar (MBBC_0.75M_) were increased by 247.2% and 46.1% compared to that of PBBC, respectively. Cd(II) adsorption by MBBC_0.75M_ was suitable to be described by the pseudo-second-order kinetic model and Freundlich isotherm. After Cd(II) adsorption, the CdCO_3_ were confirmed by XRD and observed through SEM. The weakness and shift of oxygen-containing functional groups in MBBC_0.75M_ after Cd(II) adsorption implied that those groups were complexed with Cd(II). The results showed that pyrolysis could not only eliminate banana fusarium wilt, but also prepare porous biochar with the wilt-infected banana straw. The porous biochar possessed the potential to adsorb Cd(II) pollutants.

## Introduction

Cadmium pollution has become a growing concern due to its association with human health such as cancer, renal damage, itai-itai disease, and hypertension^1–6^. Water, the primary carrier of Cd(II) into farmland, is particularly susceptible to contamination from domestic sewage and metallurgical industries^7^. Once the contaminated water infiltrates into the soil, human health can be threatened by the Cd(II) through food chain^8,9^. Therefore, controlling Cd(II) concentrations in water systems is of great importance to human been.

Biochar, serving as a porous carbon, is among the effective materials for controlling Cd(II) pollution^10–13^. Biochars derived from field crops and woody plants such as rice straw^14^, bamboo, wild cherry, walnut^15^, hardwood^16^ and so on^17,18^ have been utilized to remove Cd(II) from water bodies. Those biochars possess a certain ability to adsorbe Cd(II), which is attributed to the well-developed pore structure. However, the pore structure depends on natural element components of feedstock such as potassium, calcium, etc. Chen et al.^19^ reported that the potassium in the pokeweed could enhance the pore development in the biochar because the potassium was able to converte into gas. The carbon skeleton would be etched with the escape of the gas during pyrolysis. Zeng et al.^20^ found that circular pores in water hyacinth biochar were created due to the escape of potassium vapors from carbon skeleton. Thus, the biochar produced by these herbaceous plants has a well-developed pore structure. It seems that herbaceous plants with specific natural constituents may serve as potential raw materials to prepare porous biochar. Nevertheless, these results are not sufficient to prove that herbaceous plants, such as the straw of banana tree, can also produce porous biochar. The reason for mentioning banana straw here lies in the following two aspects. On the one hand, the banana straw represents an underutilized biomass resource^21–23^, and on the other hand, the banana straw is characterized by its high potassium content that is similar to pokeweed and water hyacinth^24^. Therefore, in order to remediate Cd(II) pollution in water bodies with porous biochar, it is necessary to study the preparation of the biochar from banana straw.

Bananas are one of the largest consumed fresh fruits in the world (FAO. 2024)^25^. Approximately 220 tons of banana straw are produced per hectare annually^26,27^. The banana straw contains significant amounts of mineral elements such as potassium, calcium, and magnesium. Those elements are able to enhance specific surface area and improve the pore structure of biochar^19,20^. Thus, banana straw has the potential to produce biochar on a large scale. Some researchers have reported the adsorption of Cd(II) by pristine biochar or KOH modified biochar derived from non fusarium wilt-infected banana straw^28,29^. However, fusarium oxysporum f. sp. Cubense Tropical Race 4 (Foc TR4) is a highly pathogenic and contagious soil-borne fungus world wide^30^. It is a common phenomenon that bananas are infected by the fusarium wilt. Once a banana is infected with Foc TR4, all organs of the plant will contain the disease^30,31^. Currently, it is not clear whether biochar prepared with fusarium wilt-infected straw still carries Foc TR4, and the adsorption effect of the KOH modified biochar prepared from the wilt-infected straw on Cd(II) remains unknown. Addressing these questions is of significant importance for the harmless utilization of the banana straw and the remediation of Cd(II) pollution. Herein, activities of the Foc TR4 in banana straw biochar were tested by physical microscope, optical microscope and double scanning laser confocal microscope. Then, Cd(II) adsorption of KOH modified banana straw biochar (MBBC) was measured. This study would expected to provide a basis for environmentally friendly utilization of banana straw and application of the banana straw biochar for remediation of Cd(II) pollution in water bodies.

## Results and discussion

### Effect of pyrolysis on viability of Foc TR4 pathogen

**Figure 1:**
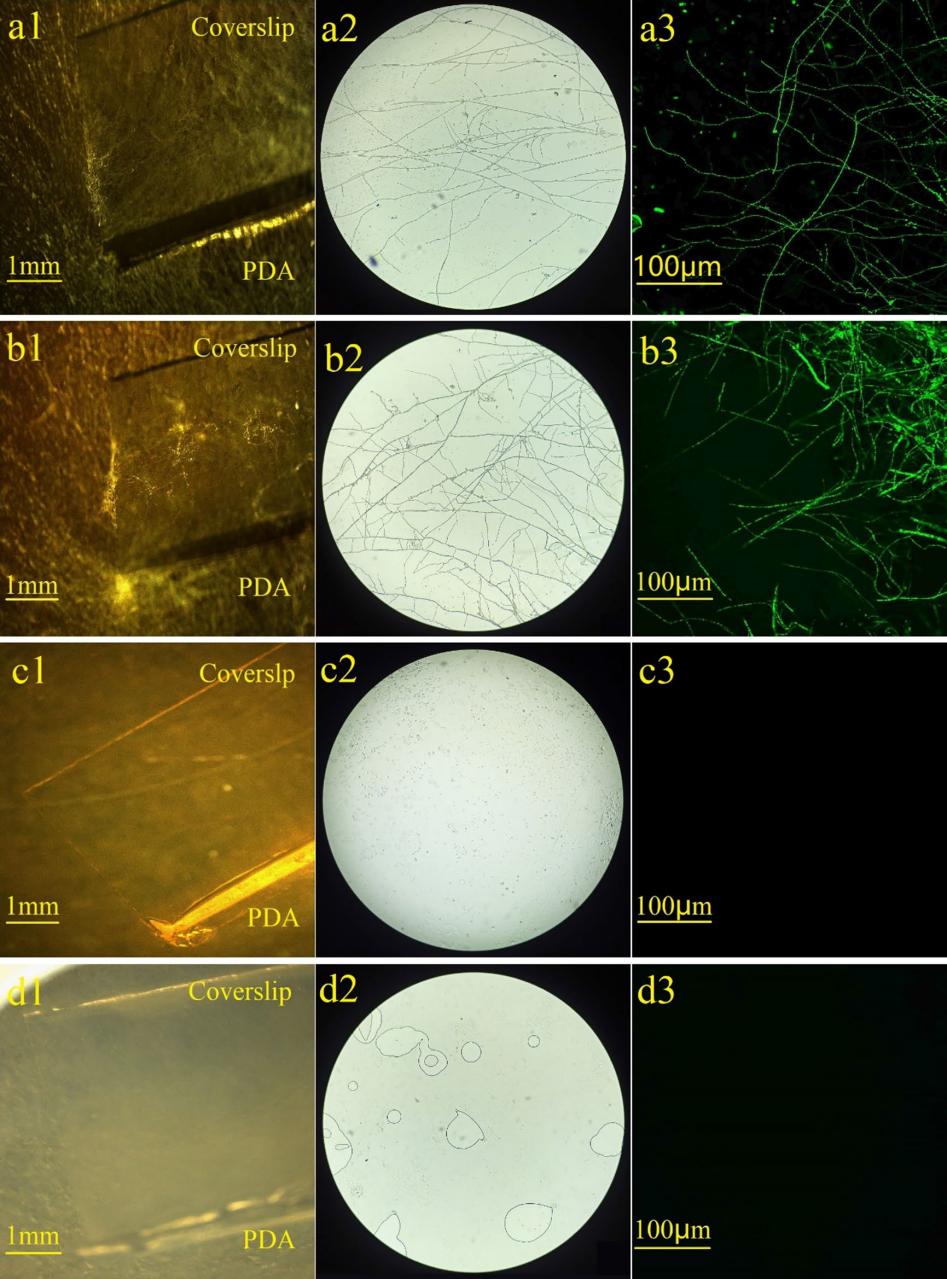
Images of spores and mycelia of Foc TR4 pathogens observed by dissecting microscopy (**a1, b1, c1, d1**), biological microscopy (**a2, b2, c2, d2**) and double-scanning laser confocal microscopy (NIKON A1) (**a3, b3, c3, d3**). (**a–d**) Treatments inoculating Foc TR4-infected banana straw, Foc TR4 pathogen, biochar prepared at 800 °C from Foc TR4-infected banana straw and healthy banana straw, respectively.

The current study showed that Foc TR4 pathogens in banana straw could be eliminated by pyrolysis. Foc TR4 mycelia were clearly visible in treatments involving inoculation with banana straw infected by the Foc TR4 pathogen (treatment 1) (Fig. 1a1 and a2) and with the Foc TR4 pathogen (treatment 2) (Fig. 1b1 and b2). A large number of mycelia were attached to the PDA plate, with some even growing on the coverslip. Additionally, the expansion and colonization of Foc TR4 conidia and mycelia in both treatment 1 and 2 were proved by the pronounced fluorescence intensity of GFP-tagged Foc TR4 in Fig. 1a3 and b3. In contrast, no mycelia were observed in treatments inoculated with biochar derived from Foc TR4 infected banana straw (treatment 3) (Fig. 1c1 and c2) or healthy banana straw (treatment 4) (Fig. 1d1 and d2). Correspondingly, no fluorescence intensity was detected in both treatment 3 and 4 (Fig. 1c3 and d3). The results confirmed that the pyrolysis effectively eliminated the Foc TR4 pathogen and ensured the safe application of banana straw biochar.

### Effect of KOH concentrations on properties and Cd(II) adsorption capacity of biochar

Numerous studies had demonstrated that the specific surface area (SSA) and heavy metal adsorption capacity of biochar could be enhanced markedly through KOH or K_2_CO_3_ modification^32–34^. As presented in Table 1, the SSA of PBBC, MBBC_0.25M_, MBBC_0.5M_ and MBBC_0.75M_ was 373.57, 821.83, 847.9 and 1297.05 m^2^ g^−1^, respectively. The pore volume was 0.24, 0.48, 0.51 and 0.82 cm^3^ g^−1^, respectively. In comparison to PBBC, the SSA of MBBC_0.25M_, MBBC_0.5M_ and MBBC_0.75M_ increased by 120%, 126.97% and 247.2%, respectively. The pore volume increased by 100%, 112.5% and 241.67%, respectively. These results confirmed that an increase in KOH concentration could significantly promote the pore development of PBBC. Moreover, micropore area of MBBC_0.25M_, MBBC_0.5M_ and MBBC_0.75M_ increased 422.36, 455.14 and 658.93 m^2^ g^−1^ compared to PBBC, all of which was higher than the external surface area. The micropore volume of MBBC_0.25M_, MBBC_0.5M_ and MBBC_0.75M_ increased by 0.21, 0.23 and 0.32 cm^3^ g^−1^, respectively, which were greater than the mesoporous volumes. Consequently, KOH modification had improved the pore structure of biochar through promoting micropore development.

**Table 1:** BET results of biochar.

Biochar samples	SSA (m^2^ g^−1^)	Micropore area (m^2^ g^−1^)	External surface area (m^2^ g^−1^)	Pore Volume (cm^3^ g^−1^)	Micropore volume (cm^3^ g^−1^)	Mesoporous volume (cm^3^ g^−1^)
PBBC	373.57	287.68	85.89	0.24	0.15	0.09
MBBC_0.25M_	821.83	710.04	111.80	0.48	0.36	0.12
MBBC_0.5M_	847.90	742.82	105.08	0.51	0.38	0.14
MBBC_0.75M_	1297.05	946.61	350.44	0.82	0.47	0.34

PBBC—pristine banana straw biochar, MBBC_0.25M_—0.25 M KOH modified banana straw biochar, MBBC_0.5M_—0.5 M KOH modified banana straw biochar, MBBC_0.75M_ —0.75 M KOH modified banana straw biochar.

**Figure 2:**
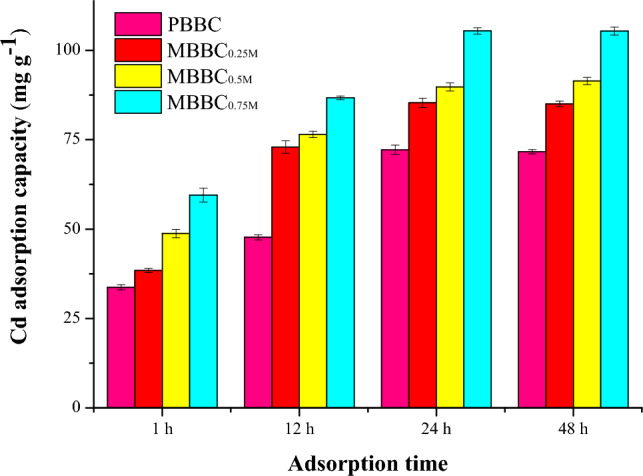
Effect of KOH concentration on the Cd(II) adsorption capacity of PBBC. Notes: PBBC—pristine banana straw biochar, MBBC_0.25M_—0.25 M KOH modified banana straw biochar, MBBC_0.5M_—0.5 M KOH modified banana straw biochar, MBBC_0.75M_ —0.75 M KOH modified banana straw biochar.

The variation in the adsorption capacity between PBBC and MBBC was illustrated in Fig. 2. The adsorption capacity of MBBC for Cd(II) increased significantly as the concentration of KOH solution raised from 0.25 to 0.75 M. The maximum adsorption capacity was reached at 24 h, after which no further increase in adsorption was observed. In comparison, the Cd(II) adsorption capacity of MBBC_0.25M_, MBBC_0.5M_ and MBBC_0.75M_ was found to be 19%, 28%, and 47% greater than that of PBBC, respectively. It could be concluded that the Cd(II) adsorption capacity of biochar was able to be raised by KOH modification. The concentration was the key factor to impact the capacity.

Table 1 and Fig. 2 showed that the optimum concentration of KOH solution was 0.75 M in the study. The specific surface area and Cd(II) adsorption capacity of MBBC_0.75M_ increased by 247.2% and 46.1%, respectively, compared to PBBC. Consequently, MBBC_0.75M_ was selected for further and deep study. The pH value, elemental composition and element ratio of MBBC_0.75M_ and PBBC were listed in Table 2. Comparatively, the pH value of MBBC_0.75M_ (12.0) was higher than that of PBBC (10.8), which implied that MBBC_0.75M_ possessed a greater ability to remediate soil pH. Obvious differences in the element contents of C, O, H and N were observed between PBBC and MBBC_0.75M_. MBBC_0.75M_ exhibited higher oxygen content but lower carbon, hydrogen, and nitrogen content compared to PBBC. The elemental composition change led to variations in the molar ratio among the elements. The H/C ratios were nearly identical, which was indicated that the high temperature during pyrolysis was sufficient to break down the original structure of banana straw. The O/C and (O + N)/C molar ratio reflected the total polar groups of biochar. MBBC_0.75M_ had significantly higher O/C and (O + N)/C ratios than PBBC, suggesting an increase in surface polar functional groups following KOH modification. In addition, the C/N ratio of MBBC_0.75M_ was approximately 79% of that of PBBC. In summary, KOH modification had improved the physicochemical properties of biochar.

**Table 2:** Physicochemical properties of PBBC and MBBC_0.75M_.

Biochars	pH	Element content %				Element ratio			
		C	H	O	N	H/C	(O + N)/C	O/C	C/N
PBBC	10.8	62.7	2.8	12.7	0.8	0.045	0.22	0.20	73.76
MBBC_0.75M_	12.0	40.4	1.8	22.6	0.7	0.046	0.58	0.56	58.55

PBBC—pristine banana straw biochar, MBBC_0.75M_ —0.75 M KOH modified banana straw biochar. H/C—ratio of hydrogen to carbon, (O + N)/C—polarity index, O/C—ratio of oxygen to carbon, C/N—ratio of carbon to nitrogen.

### Cd(II) adsorption kinetics, isotherms and thermodynamics of MBBC_0.75M_

**Figure 3:**
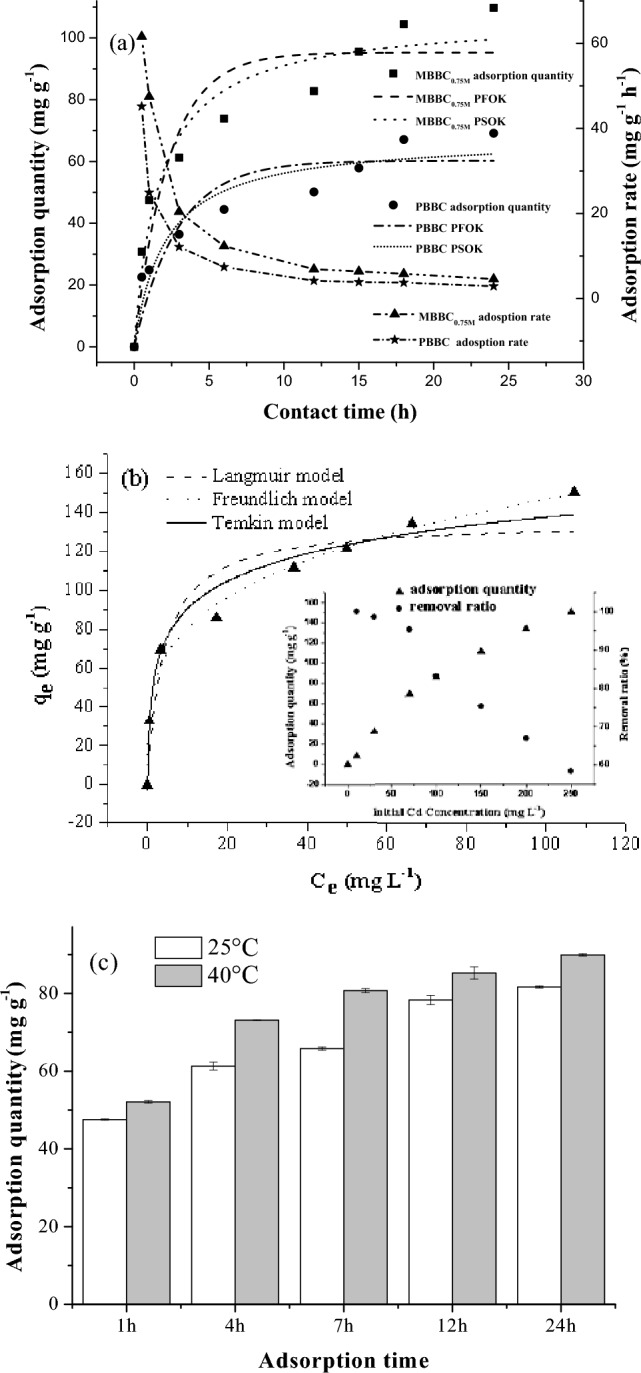
(**a**) Cd(II) adsorption kinetics of PBBC and MBBC_0.75M_. (**b**) Cd(II) adsorption isotherm of MBBC_0.75M_. (**c**) Effect of temperature on Cd(II) adsorption of MBBC_0.75M_. Notes: PBBC—pristine banana straw biochar, MBBC_0.75M_—0.75 M KOH modified banana straw biochar.

The Cd(II) adsorption kinetics of MBBC_0.75M_ and PBBC were investigated and the results were shown in Fig. 3a. The adsorption quantity of Cd(II) by both MBBC_0.75M_ and PBBC raised with increasing contact time. MBBC_0.75M_ showed a higher adsorption capacity than PBBC. The Cd(II) adsorption curve of PBBC and MBBC_0.75M_ could be divided into two sections: an initial rapid adsorption within a few hours and then enter the slow process. The relationship between Cd(II) adsorption and contact time was modeled using the pseudo-first-order kinetic (PFOK) model and pseudo-second-order kinetic (PSOK) model, respectively. The parameters of the PFOK and the PSOK were listed in Table 3. Compared to PFOK, the correlation coefficient (*r*) of PSOK in both MBBC_0.75M_ and PBBC were higher than that of PFOK, while the chi-square (*χ*^*2*^) and root mean square error (*RMSE*) were lower than that of PFOK. These results indicated that PSOK was more suitable to reveal the pattern of the Cd(II) adsorption by both the MBBC_0.75M_ and PBBC. In other word, the PSOK was suitable for fitting the relationship between Cd(II) adsorption capacity and contact time.

**Table 3:** Pseudo-first-order kinetic and pseudo-second-order kinetic parameters for Cd(II) adsorption by MBBC_0.75M_ and PBBC.

Samples	Pseudo-first-order model						Pseudo-second-order model				
	*q*_*e, exp*_	*k*_*1*_	*q*_*e, cal*_	*r*	*χ*^*2*^	*RMSE*	*k*_*2*_	*q*_*e, cal*_	*r*	*χ*^*2*^	*RMSE*
MBBC_0.75M_	109.8	0.4376	95.25	0.8803	8.98	11.47	0.0055	106.56	0.9528	3.88	7.93
PBBC	69.2	0.3485	60.26	0.8774	8.78	8.00	0.0072	67.76	0.9202	4.80	3.31

PBBC—pristine banana straw biochar, MBBC_0.75M_—0.75 M KOH modified banana straw biochar. *q*_*e, exp*_ (mg g^−1^): experimental adsorption quantity. *q*_*e, cal*_ (mg g^−1^): calculated adsorption quantity. *k*_*1*_ (mg g^−1^ min^−1^) and *k*_*2*_ (g mg^−1^ min^−1^): adsorption rate constant.

The Cd(II) adsorption isotherm of MBBC_0.75M_ was shown in Fig. 3b. The Cd(II) adsorption quantity by MBBC_0.75M_ was related to the initial Cd(II) concentration. That is, the adsorption quantity increased with the initial Cd(II) concentration during 24 h of contact at 25 °C. This trend could be attributed to the fact that a higher initial concentration of Cd(II) solution provided more opportunities for Cd(II) to interact with MBBC_0.75M_. Furthermore, the Cd(II) adsorption capacity of MBBC_0.75M_ reached 150.09 mg g^−1^ at room temperature (25 °C), which was higher than that of other adsorbents according to the reports in literature (Table 4). Conversely, the removal ratio decreased with increasing initial Cd(II) concentration (Fig. 3b). To reveal the influence of initial Cd(II) concentrations on the adsorption, the Langmuir, Freundlich and Temkin models were also employed to describe the adsorption process. The Freundlich equation was the optimum one to describe the pattern of Cd(II) adsorption by MBBC_0.75M_ because of the highest *r* (0.9956), the lowest *χ*^*2*^ (0.85) and *RMSE* (4.21) values among the three models (Table 5). This result indicated that Cd(II) adsorption by MBBC_0.75M_ was multilayer adsorption, not confined to a monolayer adsorption.

**Table 4:** Comparison of the adsorption capacity of the MBBC_0.75M_ with other carbon adsorbents for Cd(II).

Adsorbents	pH	Concentration range (mg L^−1^)	Adsorption capacity (mg g^−1^)	References
Magnetized activated carbons	6.0	10–300	73.3	^35,35^
Iron-trimeric metal–organic frameworks	5.0	10–50	8.79	^36,36^
MnO_2_ functionalized multi-walled carbon nanotubes	5.0	5–30	41.6	^37,37^
Modified coal fly ash	5.0	100–500	79.8	^38,38^
Calcium titanate particles	6.0	280–440	82.6	^39,39^
Magnetic graphene oxide composites	5.0	100–500	128.2	^40^
Citric acid and Fe_3_O_4_-modified sugarcane bagasse	6.0	0–60	33.2	^41^
MBBC_0.75M_	5.5	10–250	150.09	This work

MBBC_0.75M_ —0.75 M KOH modified banana straw biochar.

**Table 5:** Specific parameters of Cd(II) adsorption isotherms.

Isotherms	Parameters				
Langmuir	*K*_*L*_	*q*_*max,cal*_	*r*	*χ*^*2*^	*RMSE*
	0.2444	135.21	0.9130	13.63	13.83
Freundlich	*K*_*F*_	*1/n*	*r*	*χ*^*2*^	*RMSE*
	43.97	0.26	0.9956	0.85	4.21
Temkin	*K*_*T*_	*K*_*Tb*_	*r*	*χ*^*2*^	*RMSE*
	0.1208	8.17	0.9856	2.66	7.58

*K*_*L*_ and *K*_*F*_—adsorption velocity cons constant. *q*_*max, cal*_—calculate maximum adsorption quantity. *K*_*T*_—Temkin isotherm constant (kJ mol^−1^). *K*_*Tb*_—Temkin isotherm equilibrium binding constant (L mg^−1^).

The Cd(II) adsorption was influenced not only by the contact time and initial Cd(II) concentration but also by temperature. To further investigated the thermodynamics of Cd(II) sorption by MBBC_0.75M_, the effect of temperature on Cd(II) adsorption of MBBC_0.75M_ was studied and the result was shown in Fig. 3c. The result indicated that the adsorption capacity of Cd(II) increased with temperature, irrespective of the duration of adsorption. The Gibbs free energy (*ΔG*^*o*^) at 25 °C and 40 °C were − 3.48 and − 5.11, respectively, which indicated that Cd(II) adsorption was a spontaneous process. The absolute value of *ΔG*^*o*^ increased with temperature, indicating that the adsorption was an energy consuming process. The positive value of *ΔH*^*o*^ (23.25) confirmed that the adsorption was endothermic process. Thus, increasing temperature could meet the energy demands of Cd(II) adsorption.

In summary, the adsorption kinetic and isotherm of Cd(II) by MBBC_0.75M_ was more accurately represented by the PSOK and Freundlich model, respectively. Elevating the reaction temperature had been shown to augment the adsorption capacity of MBBC_0.75M_ for Cd(II). The adsorption of Cd(II) by MBBC_0.75M_ was an energy consumption process.

### Adsorption mechanism of MBBC_0.75M_ for Cd(II)

**Figure 4:**
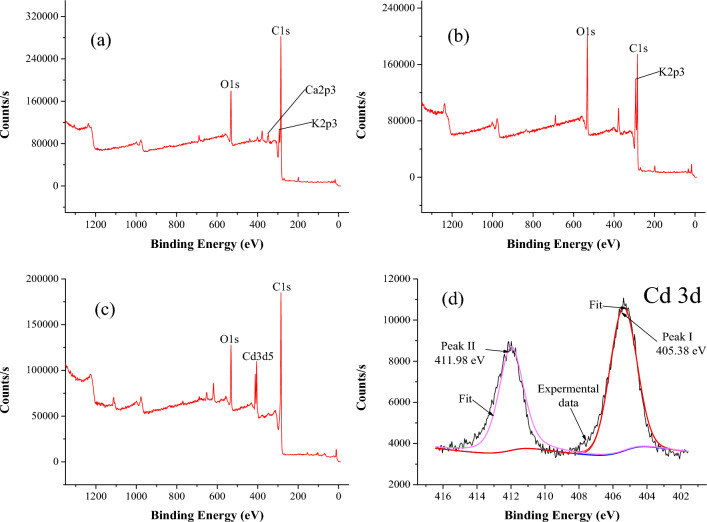
The elemental composition and combination state of PBBC (**a**), MBBC_0.75M_ (**b**) and MBBC_0.75M_A (**c**) measured by XPS. (**d**) The deconvoluted spectra of Cd 3d of MBBC_0.75M_A. Notes: PBBC—pristine banana straw biochar, MBBC_0.75M_ —0.75 M KOH modified banana straw biochar, MBBC_0.75M_A—MBBC_0.75M_ after Cd(II) adsorption.

Photoelectron spectroscopy was used to investigate the valence state of the cadmium bound on the biochar (Fig. 4). There was no cadmium peak observed in PBBC and MBBC_0.75M_ (Fig. 4a, b). However, the peak of Cd 3d was observed on MBBC_0.75M_A (MBBC_0.75M_ after Cd(II) adsorption) (Fig. 4c), which proved that Cd(II) was successfully adsorbed on the surface of MBBC_0.75M_. Figure 4d showed that the Cd 3d peak was classified into two peaks, which indicated that there existed two different combination states between Cd(II) and MBBC_0.75M_. The first peak (peak I) at 405.38 V was attributed to the precipitation of CdCO_3_^42^. The second peat at 411.98 V (peak II) was due to the complexed cadmium^43^.

**Figure 5:**
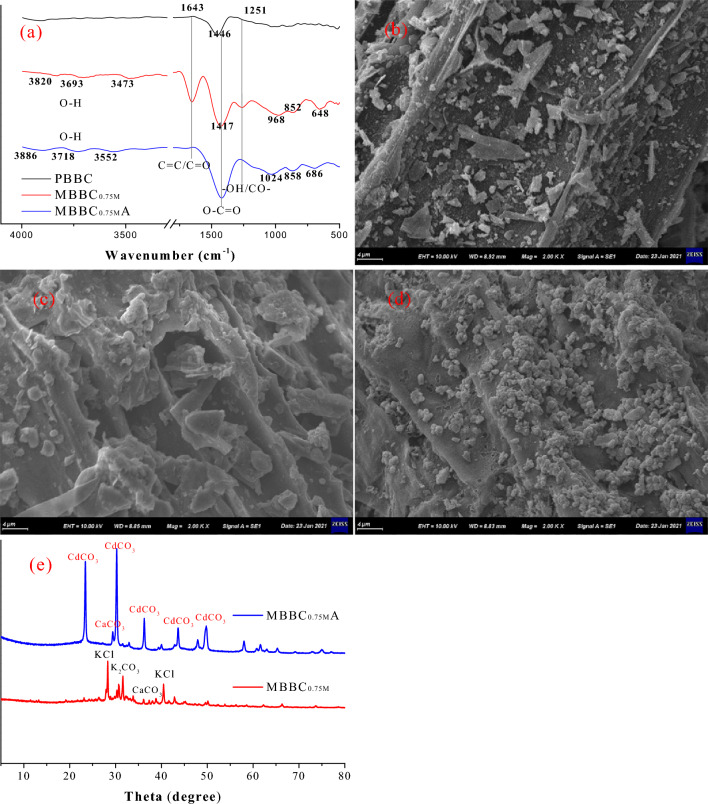
(**a**) FTIR spectra of PBBC, MBBC_0.75M_ and MBBC_0.75M_A. (**b–d**) The SEM images of PBBC, MBBC_0.75M_ and MBBC_0.75M_A, respectively. (**e**) The XRD image of MBBC_0.75M_ and MBBC_0.75M_A. Notes: PBBC—pristine banana straw biochar, MBBC_0.75M_ —0.75 M KOH modified banana straw biochar, MBBC_0.75M_A—MBBC_0.75M_ after Cd(II) adsorption.

The surface functional groups of PBBC, MBBC_0.75M_ and MBBC_0.75M_A were characterized using FTIR analysis (Fig. 5a). The peaks from 3473 to 3886 cm^−1^ were ascribed to –OH stretching of the hydroxyl group^44^. The peak at 1643 cm^−1^ could be assigned to the aromatic C=C groups or the C=O stretching of ketones, quinones, and amides^48^. Peaks observed at 1446 cm^−1^ and 1417 cm^−1^ could be assigned to O–C=O stretching vibrations of carboxylate (carboxylic acid salt)^45^. The peak at 1251 cm^−1^ could be attributed to the aromatic CO– and phenolic –OH stretching^46^. Peaks observed from 648 cm^−1^ to 1024 cm^−1^ could be due to the presence of mineral fractions^47^. In comparison to PBBC, MBBC_0.75M_ exhibited new oxygen-containing functional groups such as O–H (at 3473 to 3820 cm^−1^), C=O or C=C (at 1643 cm^−1^), and CO– and –OH (at 1251 cm^−1^). This result agreed with the increase in the O/C and (O + N)/C molar ratios of MBBC_0.75M_ (Table 2). Notably, The shift of O–H (at 3473, 3693 and 3820 cm^−1^) and the weakness of C=O or C=C (1643 cm^−1^) and CO– and –OH (1251 cm^−1^) suggested that the adsorption of Cd(II) was a complexation reaction between Cd(II) and the oxygen-containing functional groups of MBBC_0.75M_. Similar results had been reported by Lu et al.^48^ and Enniya et al.^49^. Additionally, there were no crystalline on the surface of PBBC (Fig. 5b) and MBBC_0.75M_ (Fig. 5c). The presence of crystalline particles on the surface of MBBC_0.75M_A (Fig. 5d) proved that the precipitation was one of the mechanism of Cd(II) adsorption. The XRD results of MBBC_0.75M_ and MBBC_0.75M_A were show in Fig. 5e. Upon the adsorption of Cd(II) by MBBC_0.75M_, the characteristic peaks corresponding to potassium chloride and potassium carbonate vanished, while a new peak indicative of cadmium carbonate emerged. This transformation was consistent with the crystalline structures observed in SEM images, confirming the formation of cadmium carbonate precipitates. The results substantiate that the precipitation was a pivotal pathway for MBBC_0.75M_ to adsorb Cd(II) in solution. In summary, the mechanisms of Cd(II) adsorption by MBBC_0.75M_ were both precipitation of Cd(II) on the biochar surface and complexation between Cd(II) and oxygen-containing functional groups.

## Conclusions

This study focus on the harmless utilization of fusarium wilt-infected banana straw and remediation of Cd(II) pollution by biochar derived from the straw. Results showed that the biochar derived from Foc TR4-infected banana straw was deemed safety. KOH modification could significantly improve the physicochemical properties and enhance Cd(II) adsorption capacity of the biochar. The optimum concentration of KOH modification was 0.75 M. The mechanism of Cd(II) adsorption by MBBC_0.75M_ was precipitation and complexation. The study provided a basis for biochar preparation from infected banana straw and its adsorption of Cd(II) in water bodies. Future researches should focus on the potential of biochar derived from infected plants for the remediation of Cd(II) pollution, as well as on conducting an in-depth study of the adsorption mechanisms.

## Materials and methods

### Materials and regents

Banana straw was collected from research station of Guangdong Provincial Engineering Technology Research Center of Low Carbon Agriculture and Green Inputs, SCAU. The banana straw was washed with tap water and collected, subsequently dried by 105 °C and passed through 0.85 mm sieve for later use.

Potassium hydroxide (KOH) was industrial grade chemical with a purity of 99.5%. The remaining reagents were of analytical grade, procured from Fuchen (Tianjin) Chemical Reagents Co., Ltd. and Guangzhou Chemical Reagents Co., Ltd.

### Biochar preparation

The banana straw was subjected to pyrolysis in a programmable tubular carbonization reactor (Tianjin Taisite Instrument Co., Ltd., China) for pyrolysis. The banana straw was heated at a rate of 3 °C min^−1^ to a final temperature of 800 °C and kept for 2 h. The biochar was collected when the reactor was cooled to room temperature and it was designated as pristine banana straw biochar (PBBC).

The carbon material prepared in this paper was modified biochar, rather than activated carbon. The KOH modified banana straw biochar (MBBC) was produced as follow. First, the banana straw was impregnated with 0.25, 0.5 and 0.75 M KOH solutions for 24 h, respectively. Then, the straw was filtered through a 0.075 mm nylon mesh to expel excess solution, followed by drying at 105 °C to obtain the modified banana straw. The gravimetric method was employed to ensure that an equal volume solution was absorbed per unit straw. Finally, the modified banana straw was pyrolyzed at 800 °C for 2 h, yielding modified biochars were named MBBC_0.25M_, MBBC_0.5M_ and MBBC_0.75M_, respectively.

### Detection of Foc TR4 spores and their viability in biochar

To validate the effectiveness of pyrolysis in eliminating the Foc TR4 pathogen, the presence and viability of the pathogen spores in biochar were determined by PDA (Potato Dextrose Agar) petri dish culture experiments. The study comprised four treatments, each representing a different source of pathogen: treatment 1 (GFP-tagged Foc TR4-infected banana straw), treatment 2 (GFP-tagged Foc TR4, representing the pure pathogen), treatment 3 (biochar prepared at 800 °C from Foc TR4-infected banana straw), and treatment 4 (normal banana straw, serving as a control). Each source of the pathogen was inoculated onto PDA plates in 90 mm petri dishes. A 24 mm × 50 mm coverslip was inserted at a 45-degree angle into the PDA medium, 15 mm away from the inoculum. The dishes were incubated at 25 °C for 5 days. The presence and viability of Foc TR4 spores and mycelia were examined using a real object microscope, an optical microscope, and a double-scanning laser confocal microscope (DSLCM, NIKON A1), respectively.

### Properties and morphology of the biochar

The surface morphology of the biochar was observed via SEM (Scanning Electron Microscopy, CARL ZEISS EVO 10, Germany) under 10.0 kV voltages. The C, H, O, and N contents were determined using an elemental analyzer (Vario EL cube, Elementar, Germany). The specific surface area (S_BET_), average pore diameter (PD) and pore volume (PV) were measured by N_2_ adsorption at 77 K with a Brunauer‒Emmett‒Teller (BET) specific surface area analyzer (ASAP 2020, USA). X-ray photoelectron spectroscopy (XPS) (Thermo Fisher Scientific K-Alpha, ThermoFisher, USA) was used to measure the elemental composition and combination state of the biochar. The surface functional groups were detected using Fourier transform infrared (FTIR) spectroscopy (Vertex 70, Bruker, Germany) in the range of 4000 to 400 cm^−1^ using KBr pellets at 25 ± 1 °C.

### Batch adsorption experiment

The effect of KOH concentration (0.25, 0.5 and 0.75 M) on the Cd (II) adsorption were investigated by adding 0.1500 g biochar (PBBC, MBBC_0.25M_, MBBC_0.5M_ and MBBC_0.75M_) to 60 mL of Cd(II) solution (350 mg L^−1^) in a 100-mL plastic vials, respectively. The solution pH was adjusted to 5.5 by NaOH (0.2 mol L^−1^) and HCl (0.2 mol L^−1^). Then these plastic vials were shaken using a thermostatically controlled shaker (TS-2102C) at 200 rpm for given time intervals. Three vials as three replications of each treatment were collected at time. The Cd(II) concentration in the filtrate of each vial was diluted with Milli-Q water, and the pH of the diluted solution was adjusted to pH 2 with 10% nitric acid. Cd(II) content was determined by atomic absorption spectroscopy (AAS) (AA-7000, Shimadzu, Japan). The adsorption capacity at equilibrium (*q*_*e*_) (Eq. 1) and removal ratio (*R%*) (Eq. 2) were calculated as follows Xu et al. (2021)^50^.

1 $${q}_{e}=({C}_{0}-{C}_{e})\times (\frac{V}{M})$$

2 $$R\%=\frac{\left({C}_{0}-{C}_{e}\right)}{{C}_{0}}\times 100$$

where *C*_*0*_ (mg L^−1^) and *C*_*e*_ (mg L^−1^) were the initial and equilibrium concentrations of Cd(II) in the solution, respectively. *M* was the weight of biochar (g). *V* was the solution volume (L).

For the adsorption kinetic study, 0.1500 g of biochar and 60 mL of Cd(II) solution (350 mg L^−1^) were added to a 100-mL plastic vials and the pH was adjusted to 5.5. Then these plastic vials were shaken at 200 rpm. At given time intervals, the solution was taken out, and the residual Cd(II) was measured by AAS. Pseudo-first-order kinetic (PFOK) model (Eq. 3) and pseudo-second-order kinetic (PSOK) model (Eq. 4) were used to fit the experimental data as follows Liang et al. (2019)^51^.

3 $$\mathit{ln}\left({q}_{e}-{q}_{t}\right)= ln{q}_{e}{- K}_{1}t$$

4 $$\frac{t}{{q}_{t}}= \frac{1}{{K}_{2}{q}_{e}^{2}}+ \frac{t}{{q}_{e}}$$

where *q*_*e*_ (mg g^−1^) was adsorption capacity at equilibrium. *q*_*t*_ (mg g^−1^) was amount of Cd(II) adsorbed at time *t*. *k*_*1*_ (mg g^−1^ min^−1^) and *k*_*2*_ (mg g^−1^ min^−1^) were adsorption rate constant of the PFOK and PSOK, respectively.

Adsorption isotherms were tested by eight initial Cd(II) concentration of 10, 30, 70, 100, 150, 180, 200 and 250 mg L^−1^, respectively. Each 0.0500 g MBBC_0.75M_ were put in twenty four 100-ml plastic vials. 50 ml each of the eight initial Cd(II) solution was added to three vials, respectively. The solution pH of each vial was adjusted to 5.5 and then the vials were shaken at 25 °C constant temperature for 24 h. Three samples from each of the eight Cd(II) solution treatment were collected at the end of the shaken. Cd(II) content in filtrate was tested as above.The adsorption isotherms were fitted with Langmuir, Freundlich and Temkin models, respectively, to further analyze the adsorption procedure of biochar for Cd(II) and to determine the maximum adsorption capacity.

Langmuir model was described as Lima et al. (2015)^52^ (Eq. 5).

5 $$\frac{{C}_{e}}{{q}_{e}}= \frac{1}{{K}_{L}{q}_{m}}+ \frac{{C}_{e}}{{q}_{m}}$$

where *C*_*e*_ (mg L^−1^) and *q*_*e*_ (mg g^−1^) were the concentration and adsorption capacity at the equilibrium. The *q*_*m*_ was maximum adsorption capacity (mg g^−1^). *K*_*L*_ was the Langmuir equilibrium constant.

The Freundlich model was described as Wang et al.^53^ (Eq. 6).

6 $$ln{q}_{e}=ln{K}_{F}+ \frac{ln{C}_{e}}{n}$$

where *K*_*F*_ was the equilibrium constant. *n* was the Freundlich exponent (dimensionless). *C*_*e*_ (mg L^−1^) and *q*_*e*_ (mg g^−1^) were the concentration and adsorption capacity at the equilibrium.

The form of Temkin isotherm model was given by the Pursell et al. (2012)^54^ (Eq. 7):

7 $${q}_{e}=\frac{RT}{{K}_{T}}ln{K}_{Tb}{c}_{e}$$

Its linear form is as follows.

$${q}_{e}=\frac{RT}{{K}_{T}}ln{K}_{Tb}+\frac{RT}{{K}_{T}}ln{c}_{e}$$

where *q*_*e*_ was adsorption amount at equilibrium (mg g^−1^); *R* was the universal gas constant (8.314 J mol^−1^ K^−1^), *T* was the absolute temperature (298.15) in K; *K*_*Tb*_ was Temkin isotherm equilibrium binding constant (L mg^−1^), *K*_*T*_ was Temkin isotherm constant (kJ mol^−1^); *C*_*e*_ was the equilibrium constant.

Thermodynamic parameters provide information regarding the inherent energetic changes involved during Cd(II) adsorption of biochar. Thirty parts of each 0.0500 g of dried MBBC_0.75M_ were weighed and put in 100-ml plastic vials, respectively. 50 mL of Cd(II) solution (100 mg L^−1^) was added to each vial. The solution pH of each vial was adjusted to 5.5 as described above. Then every 15 vials were shaken at a rate of 200 r min^−1^ under 25 °C and 40 °C, respectively. Three samples were taken at given time intervals, respectively. Then, the concentration of Cd(II) in the filtrate was tested by AAS. Thermodynamic parameters were calculated by following Archana et al. (2007)^55^. The changes in thermodynamic parameters of standard Gibbs free energy (*ΔG*^*o*^), standard enthalpy (*ΔH*^*o*^) and standard entropy (*ΔS*^*o*^, J mol^−1^ K^−1^) were calculated from the variation of the thermodynamic equilibrium constant, *K*_*0*_, with temperature change. *ΔG*^*o*^ (kJ mol^−1^), *ΔH*^*o*^ (kJ mol^−1^) and *ΔS*^*o*^ (J mol^−1^ K^−1^) were calculated using Eqs. (8) and (9).

8 $${G}^{o}= -RT\mathit{ln}{K}_{0}$$

9 $$\mathit{ln}{K}_{0}= \frac{\Delta {S}^{o}}{R}- \frac{\Delta {H}^{o}}{RT}$$

Thermodynamic constant, *K*_*0*_, for the adsorption reaction at equilibrium can be defined as Eq. (10) when the concentration of the Cd(II) in the solution approaches zero.

10 $${K}_{0}= \frac{{a}_{s}}{{a}_{e}}= \frac{{q}_{e}}{{C}_{e}}$$

where *a*_*s*_ and *a*_*e*_ denoted activity coefficients of the Cd(II) adsorbed onto the MBBC and Cd(II) ions in the equilibrium solution, respectively. *C*_*e*_ (mg L^−1^) and *q*_*e*_ (mg g^−1^) were the concentration and adsorption capacity at the equilibrium, respectively.

The goodness of fit between the experimental data and model predicted values was expressed by the correlation coefficient (*r*) and chi-square (*χ*^*2*^) and root mean square error (*RMSE*). The *χ*^*2*^ and *RMSE* were expressed as Eqs. (11) and (12)^40,56^.

11 $${\chi }^{2}=\sum_{i=1}^{n}\frac{{({q}_{e,exp} - {q}_{e,cal})}^{2}}{{q}_{e,cal}}$$

12 $$RMSE=\surd {\sum }_{i=1}^{n}\frac{{({q}_{e,cal} - {q}_{e,exp})}^{2}}{n}$$

where *q*_*e,exp*_ was amount of Cd(II) adsorption at equilibrium in the experiment and *q*_*e,cal*_ was calculated. The larger the *r* and the smaller the *χ*^*2*^ and *RMSE*, the higher the goodness of fit.

### Plants material policy

The authors solemnly undertake that the collection process of banana straw and other experimental materials used in this study complied with the regulations of South China Agricultural University and the Chinese government. The materials comply with the IUCN Policy Statement on Endangered Species Research and the Convention on Trade in Endangered Species of Wild Fauna and Flora.

## Data Availability

Data will be made available on request. Please send email to chengxianggao@163.com for research data in this paper.
